# Mifepristone for Treatment of Metabolic Syndrome: Beyond Cushing’s Syndrome

**DOI:** 10.3389/fphar.2020.00429

**Published:** 2020-04-24

**Authors:** Francisco Díaz-Castro, Matías Monsalves-Álvarez, Leonel E. Rojo, Andrea del Campo, Rodrigo Troncoso

**Affiliations:** ^1^ Laboratorio de Investigación en Nutrición y Actividad Física (LABINAF), Instituto de Nutrición y Tecnología en Alimentos (INTA), Universidad de Chile, Santiago, Chile; ^2^ Advanced Center for Chronic Diseases (ACCDIS), Universidad de Chile, Santiago, Chile; ^3^ Departamento de Biología, Facultad de Química y Biología, Universidad de Santiago de Chile, Santiago, Chile; ^4^ Centro de Biotecnología Acuícola, Universidad de Santiago de Chile, Santiago, Chile; ^5^ Departamento de Farmacia, Facultad de Química y de Farmacia, Pontificia Universidad Católica de Chile, Santiago, Chile

**Keywords:** RU486, insulin resistance, skeletal muscle, mitochondria, glycaemia

## Abstract

A growing body of research indicates that cortisol, the glucocorticoid product of the activation of the hypothalamic-pituitary-adrenal axis, plays a role in the pathophysiology of metabolic syndrome. In this regard, chronic exposure to cortisol is associated with risk factors related to metabolic syndrome like weight gain, type 2 diabetes, hypertension, among others. Mifepristone is the only FDA-approved drug with antiglucocorticoids properties for improved the glycemic control in patients with type 2 patients secondary to endogenous Cushing’s syndrome. Mifepristone also have been shown positive effects in rodents models of diabetes and patients with obesity due to antipsychotic treatment. However, the underlying molecular mechanisms are not fully understood. In this perspective, we summarized the literature regarding the beneficial effects of mifepristone in metabolic syndrome from animal studies to clinical research. Also, we propose a potential mechanism for the beneficial effects in insulin sensitivity which involved the regulation of mitochondrial function in muscle cells.

## Introduction

Mifepristone, also known as RU486, was the result of the biomedical revolution forced by the wave of women´s movements in the 20^th^ century seeking to control their reproductive life (Spitz, 2010). This drug is a synthetic hormone with antiprogestogen receptor blocker and glucocorticoid receptor (GR) antagonism activity. However, their first and most common use is as an emergency contraceptive or abortion-inducing pill during the first month of pregnancy (Rodger and Baird, 1987; Silvestre et al., 1990). Mifepristone had also demonstrated to display antiproliferative and antimetastasis effects in several cancer cell lines such as breast, endometrium, cervix, prostate, gastrointestinal tract, brain, bone, and ovary (Chen et al., 2014).

Regarding the antiglucocorticoid properties, mifepristone has three and 10 times higher relative binding affinity for the GR than dexamethasone and cortisol, respectively (Castinetti et al., 2012). In general terms, glucocorticoids regulate numerous physiological processes that maintain the organism’s homeostasis (Ramamoorthy and Cidlowski, 2013). However, chronic exposition to endogenous and exogenous glucocorticoids is associated with the appearance of several signs or diseases such as hyperglycemia, weight gain, hypertension, type 2 diabetes, muscle weakness, osteoporosis, depression, and decreased immune function (Schäcke et al., 2002). Due to the several effects caused by chronic glucocorticoids exposure, mifepristone is being investigated as a potential therapeutic drug for the treatment of psychiatric disorders such as psychotic depression, alcohol and cocaine dependence, schizophrenia, and bipolar disorder (Howland, 2013). There has also been reported one successful case of mifepristone use is the control of glycaemia in patients with Cushing’s syndrome (Fleseriu et al., 2012). Due to the favorable results found in glycaemia control in these patients, the U.S. Food and Drug Administration (FDA) approved in 2012 the use of mifepristone for glycaemia control in patients with Cushing’s syndrome who have developed type 2 diabetes (Korlym^®^). Moreover, since 1995 to date several studies in rodents and humans with metabolic syndrome had shown beneficial effects of mifepristone (see **Table 1**). Despite the accumulative evidence, little is known about the mechanisms involved in the hypoglycemic effects of mifepristone. Here, in this perspective article, we briefly summarize the available evidence on the beneficial effects of mifepristone in Cushing’s syndrome and metabolic syndrome and, propose a feasible mode of action that involves the regulation of mitochondrial function, which could explain mifepristone insulin-sensitizing effects.

**Table 1:** Mifepristone effects in model of metabolic syndrome.

Reference	Model	Mifepristone doses/via administration/treatment time	Insulin sensivity effect	Glycemic effect	Weight Changes	Other effects	Comments
Kusunoki et al., 1995	High fat diet and high starch diet (rats)	30 mg • kg-1 • day-1/In the food/25 days	**↑**	**=**	**=**	N/A	Mifepristone reverse the insulin resistance induced by HFD. In skeletal muscle, mifepristone produced the major effect.
Gettys et al., 1997	ob/ob mice	30 mg • kg • day/subcutaneously injection/21 days	N/A	**↓**	**=**	N/A	Mifepristone reduce fasting glucose amd insulin levels in ob/ob mice.
Taylor et al., 2009	ob/ob mice	30 mg • kg • day/subcutaneously injection/28 days	**↑**	**↓**	=	N/A	–
Gross et al., 2009	Olanzapine traetment (human)	600 mg • day/oral/14 days	=	=	**↓**	↓ Waist circumference ↓ BMI	Mifepristone reduced weight gain.
Gross et al., 2010	Risperidone traetment (human)	600 mg • day/oral/28 days	**↑**	N/A	**↓**	↓Triglycerides ↓Food consumption	Mifepristone reduced HOMA-IR.
Hashimoto et al., 2013	High fat diet (mice)	0.1 - 1.0 and 30 mg • kg • day/Orally/22 weeks	**↑**	**↓**	**=**	↑ adiponectin secretion ↓ Liver weight ↓ Adipocity area ↓ Aspartate aminotransferase	The higher effects were seen with a dose of 30mg • kg • day of mifepristone
Nikolic et al., 2013	MIF-KO mice	20 mg • kg • day/N/A/7 days	**↑**	**↓**	N/A	N/A	Mifepristone increased the phosphorylation induced by insulin in liver.
van den Heuvel et al., 2016	High fat fructose diet (mice)	60 mg • kg • day/in the food/4 weeks	N/A	**↓**	**↓**	↓ fat mass	Mifepristone reduced the inhibition of lipolysis by insulin
Priyadarshini and Anuradha, 2017	High fructose diet (mice)	20 mg • kg • day/oral gavage/18 days	N/A	**↓**	**↓**	↓ Liver weight ↓Viceral adipose tissue ↑ Liver triglycerides ↓ Total cholesterol ↓ Free fatty acids	Mifepristone reduced GC-target genes in liver.
Bernal-Sore et al., 2018	L6 myotubes (rat)	10 µM/24 h	**↑**	**↓**	N/A	↓Oxygen consumption ↓Intracellular ATP ↓ROS production	The RU486 increase insulin sensitivity through AMPK activation

↑ increase; ↓ decrease; = not change; N/A not assessed.

## Mifepristone In Cushing’s Syndrome

Cushing’s syndrome (CS) is a condition where cortisol plasma levels are high for a long period of time, developing different signs and symptoms, such as weight gain (especially in the upper body), rounded face, insulin resistance, hypertension, osteoporosis and/or muscle weakness (Newell-Price et al., 2006). This deregulation may be produced by endogenous or exogenous factors. The less common is the endogenous CS, which is usually caused by a tumor that produces an excess of cortisol or ACTH depending on its anatomical location (adrenal or pituitary gland) (Allolio and Fassnacht, 2006). The most common cause for CS is the treatment with exogenous glucocorticoids, such as prednisone, for asthma, rheumatoid arthritis, and immunosuppression organ transplantation (Lacroix et al., 2015).

During 2011 the results from the clinical trial “A Study of the Efficacy and Safety of CORLUX (now registered as Korlym®) for the Treatment of Endogenous Cushing’s Syndrome (SEISMIC)” (NCT00569582) were published. This study enrolled 50 patients for 24 weeks with endogenous CS. The results showed that subjects treated with mifepristone had improvement of physical parameters such as body weight, fat mass, waist circumference and also improvement of insulin sensitivity in 87% of patients. In subjects with diabetes mellitus or glucose intolerance, the improvement in glucose profiles was of 60% (Fleseriu et al., 2012). Before that, other works reported benefits of mifepristone use in patients with CS. In 1985, Neiman et al., treated successfully a patient with CS due to ectopic ACTH secretion. The patient improved somatic features as buffalo hump, central obesity and moon face, and sensitive glucocorticoid parameters such as fasting blood glucose (Nieman et al., 1985). Other cases of patients with CS treated with mifepristone have been summarized in a previous retrospective study. The authors concluded that treatment with mifepristone produced a reduction in signs of hypercortisolism, half-reduced blood pressure and half of the patients with diabetes improved blood glucose levels (Castinetti et al., 2010). In 2015, the follow-up and long-term extension of the SEISMIC clinical trial (NCT00936741) was published. In this report, the weight loss achieved during the treatment of 24-week in SEISMIC, persisted for two additional years in patients who remained on therapy (Fein et al., 2015). Due to the results obtained in SEISMIC, Korlym® was FDA-approved for their use in patients with type 2 diabetes or glucose intolerance secondary to endogenous CS. In addition, to the beneficial effects on glycaemia control in patients with CS, several works have reported beneficial effects of mifepristone in metabolic syndrome (see **Table 1**).

## Mifepristone in Metabolic Syndrome

The metabolic syndrome is a group of physiologic, biochemical and metabolic factors that lead to increased risk for type 2 diabetes, stroke and cardiovascular diseases (Eckel et al., 2005). These factors (visceral obesity, dyslipidemia, hyperglycemia and hypertension) are associated with changes in lifestyle, mainly in dietary preferences and sedentary behavior (Dalle Grave et al., 2010). Insulin resistance is a major underlying mechanism of metabolic syndrome, which affects different organs such as the brain, liver, pancreas, vascular endothelium, adipose tissue, heart and skeletal muscle, which contribute to development of metabolic syndrome (Guo, 2014). People with metabolic syndrome may have abnormal levels of cortisol, as in Cushing patients, but without developing CS (Pasquali and Vicennati, 2000). Whereby the deregulation of cortisol action could have an important role in the development of metabolic syndrome, but this is still uncertain (Paredes and Ribeiro, 2014). Mifepristone, being the only antiglucocorticoid drug clinically available, has been used to investigate this hypothesis and has promise as a potential insulin sensitizer (see **Table 1**). In 1995, Kusunoki et al. showed the first approximation in the metabolic effects of mifepristone independently of CS, where animals fed with a high-fat diet and treated with mifepristone showed an improvement in insulin sensitivity without changes in body weight (Kusunoki et al., 1995). Moreover, mifepristone enhances insulin-dependent glucose uptake, improving insulin sensitivity in obese animals (Hashimoto et al., 2013). In addition, mifepristone ameliorates diabetes symptoms in ob/ob mice (Gettys et al., 1997), improves glucose tolerance in knockout mice for the macrophage migration inhibitory factor (Nikolic et al., 2013), decreases lipid abnormalities and reduces insulin resistance in mice fed with high fructose diet (Priyadarshini and Anuradha, 2017). Moreover, mifepristone ameliorates obesity and metabolic perturbations in patients with antipsychotic medication (Gross et al., 2009; Gross et al., 2010). All of these reports pointed that mifepristone could be useful in the treatment of metabolic syndrome not associated with CS, however the mechanisms of the beneficial action of mifepristone in metabolic syndrome are far from being elucidated.

As is known, one of the most important tissues in glycemic control is the skeletal muscle, where approximately 80% of the glucose uptake is carried out and used in postprandial state (Otto Buczkowska and Dworzecki, 2003). Skeletal muscle insulin response is one of the mayor processes altered in metabolic syndrome, thus leading to high plasmatic levels of glucose and insulin resistance (Guo, 2014). In this regard, we recently reported a potential mechanism the beneficial effects of mifepristone associated with skeletal muscle physiology. We demonstrated that mifepristone increases insulin-dependent glucose uptake through a mitochondrial-AMPK pathway (Bernal-Sore et al., 2018).

## Mitochondrial Function as a Target of Mifepristone

Mitochondria are known as the powerhouse of the cell by its main role in ATP production, but it is also important for other processes, such as signaling pathways (Goldenthal and Marín-García, 2004), cell cycle regulation (Finkel and Hwang, 2009), oxidative stress (Orrenius et al., 2007), and apoptosis (Xiong et al., 2014) among others. This organelle is able to synthesize ATP through oxidative phosphorylation (OXPHOS) which uses reducing agents (NADH-FADH_2_) from the oxidation of glucose, fatty acids, and ketone bodies on Krebs’s cycle, counting for almost 90% of the energy produced by the cell (Senior, 1988).

Mitochondrial function and response to stimuli are highly dependent on their structure and dynamics. Mitochondria can modulate their morphology in response to stressors to create an elongated mitochondrial network or fragmented mitochondria depending on energy demands (Eisner et al., 2018). These changes on shape and distribution of mitochondria are known as mitochondrial dynamics and are controlled by fusion events, that depend on Mitofusins (MFN1 and MFN2), which catalyze the outer membrane fusion, and the optic atrophy 1 gene (OPA1) in charge of the inner membrane fusion, and by fission, which needs the recruitment of Dynamin-related protein 1 (DRP1), by fission 1 homolog 1 (FIS1), and mitochondrial fission factor (MFF), to the outer membrane to constrict the mitochondria and divide into two uneven daughters mitochondria (Sebastián et al., 2017).

Mitochondrial dysfunction has gained attention as an important player in the development of type 2 diabetes, obesity, dyslipidemia, and cardiovascular diseases. Excess in ROS production or nutrient supply led to a decrease of mitochondrial protein levels, changes in substrate oxidation and modifications on the shape and size of the mitochondria (Joseph et al., 2012). In addition, changes on the expression of mitochondrial dynamics related proteins in type 2 diabetes have been reported, particularly a downregulation of MFN2 and an increase on DRP1, favoring mitochondrial fission (Joseph et al., 2012).

Our recent research demonstrated that mifepristone enhances insulin-stimulated glucose uptake on L6 skeletal muscle cells through a mechanism that reduces oxygen consumption, ROS levels and ATP production, promoting AMPK activation (Bernal-Sore et al., 2018). The activation of AMPK, subsequent to the decrease of ATP production, has been previously documented as a possible mechanism of several compounds that promote insulin sensitivity (Smith and Steinberg, 2017). These compounds include berberine, a herb-derived drug widely used in China for the treatment of type 2 diabetes (Yin et al., 2008), metformin which inhibits the respiratory chain complex I (Brunmair et al., 2004; Martineau, 2012) and resveratrol, which inhibits the mitochondrial ATP synthase (Zang et al., 2006). In the case of mifepristone, the mechanism associated with the regulation of mitochondrial function is not yet elucidated.

**Figure 1:**
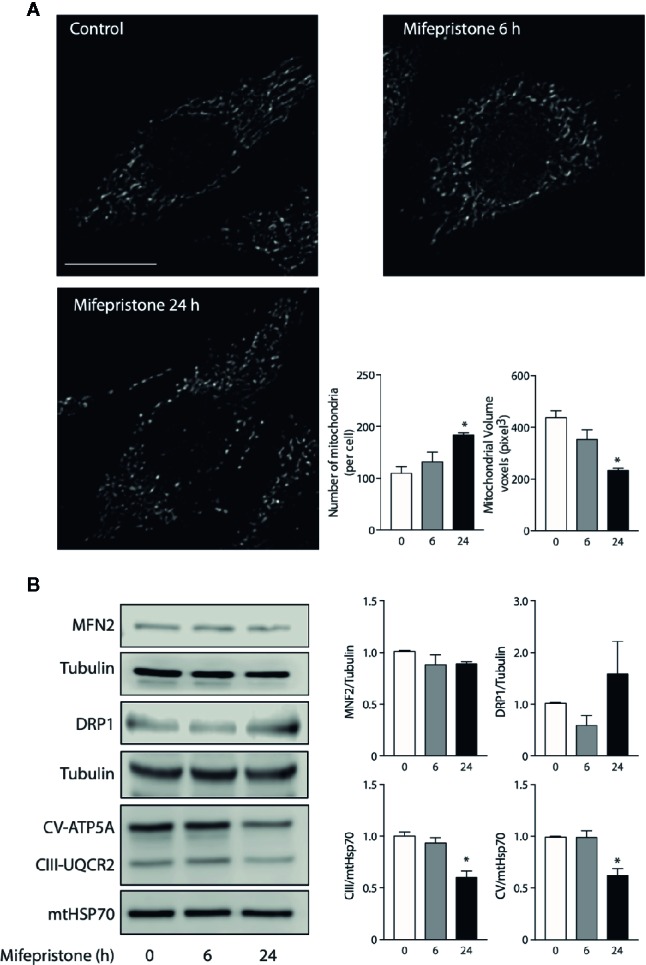
Mifepristone targets mitochondria. **(A)** Time course of mifepristone on mitochondrial morphology. Cells were incubated with mifepristone (10 uM) at indicated times and then loaded with MitoTracker Orange. Multislice imaging reconstitution was obtained by confocal microscopy. The scale bar is 10 μm. The individual mitochondrial volume and number of mitochondria per cell were determined. **(B)** Left panel: Representative immunoblot of the effect of mifepristone treatment in MNF2, DRP1, CV-ATP5A, CIII-UQCR2, mtHSP70, and tubulin protein levels; Right panel: Densitometry analysis. (mean ± SEM; n 4). *p < 0.05 versus control.

To gain insight into how mifepristone affects mitochondrial function, we evaluated the changes in mitochondrial morphology. To assess this, we incubated L6 myoblast with Mitotracker Orange after incubation with mifepristone 10 μm for 6 and 24 h and fixed with PFA 4% to subsequently mount them in a coverslip. Confocal images stack of the mitochondrial network were captured with a Nikon C2 Confocal microscope. The number and volume of individual mitochondria were quantified using ImageJ software (NIH) as described previously (del Campo et al., 2014). Our results show that mifepristone induces an increase in the number and a decrease in the volume of mitochondria at 6 and 24 h of treatment when compared to control cells, which determine a fragmented phenotype (**Figure 1A**). Then, we evaluate the protein levels of MFN2 and DRP1. Our results show that mifepristone induces a non-statistical increase in DRP1 without changes in MFN2 protein levels, which could be associate with an increase in mitochondrial fragmentation (**Figure 1B**) (Sebastián et al., 2017). These results suggest that mifepristone regulates mitochondrial morphology toward a fragmented state, which correlates with the decrease in mitochondrial function that we previously reported (Bernal-Sore et al., 2018). Fusion and fission processes are part of the mitochondria life cycle, which allow the mitochondria to respond to cell stressors adapting ATP production efficiency (Liesa and Shirihai, 2013). To further elucidate whether changes in mitochondrial morphology correlate with mitochondrial function, we examined the modification in OXPHOS proteins levels. Our results show that mifepristone reduces the protein levels of mitochondrial respiratory chain complexes III and V (**Figure 1B**). Thus, it is possible that mifepristone-induced OXPHOS reduction leads to fragmentation of the mitochondrial network due to a compensatory effect triggered by the reduction in ATP levels and an increase in AMPK activity (Toyama et al., 2016). However, how mifepristone is affecting mitochondria is still unknown, the aforementioned results point this could be through a direct effect on mitochondria, just as metformin or resveratrol, without discarding the effects of its antagonist activity over the GR. Regarding the latter, it is known that glucocorticoids regulate mitochondrial RNA synthesis (Mansour and Nass, 1970). Moreover, GR localized inside the mitochondria can regulate OXPHOS by binding to mitochondrial DNA and activating mitochondrial gene expression in HepG2 cells (Psarra and Sekeris, 2011). The above, open the possibility that mifepristone may regulate mitochondrial function through the control of mitochondrial gene expression.

## Concluding Remarks

The beneficial effects of mifepristone in the control of glycaemia in patients with CS had been proven. In addition, several clinical and basic research works have reported its potential use as an insulin sensitizer in different models of metabolic syndrome (**Table 1**). However, the mechanisms of these beneficial effects are not completely elucidated. Here, we propose a new perspective to explore the mechanism of mifepristone-induced insulin sensitization based on the regulation of OXPHOS and mitochondrial dynamics, which could explain the reduction in ATP levels and the activation of AMPK. In this regard, mifepristone improves glycemic control through the regulation of mitochondrial function in skeletal muscle, but its feasible to propose that mifepristone also could control insulin resistance in other organs. Moreover, in patients with CS mifepristone decreases body weight, fasting glucose and blood pressure which suggests that other tissues, like brain, liver and endothelium could be involved in the beneficial effects of mifepristone (Fleseriu et al., 2012). It is important to note, that the consequences of mifepristone in glycemic control are similar to approved drug for diabetes such as pioglitazone and metformin, which mechanism of action also is related in regulation of AMPK activity and mitochondrial function, but mifepristone may have a subtly different mechanism of action or side-effects profile that could be helpful in pool of patients with different base pathology, risk factors or response to treatment.

Nonetheless, the first use of mifepristone was as a contraceptive drug. Therefore, the use of this drug should be restricted in some populations, including pregnant women and women who wish to get pregnant. Mifepristone’s other potential side-effects should also be considered. Furthermore, effects in fertility and endocrine system after a short and long-term treatment should be studied to determine the potential patients of this drug and thus be able to determine the potential advantages of mifepristone or mifepristone-derived drugs over currently available treatments for metabolic disorders.

## Data Availability Statement

The datasets generated for this study are available on request to the corresponding author.

## Author Contributions

FD-C performed experiments, data analysis, and contributed to the manuscript writing. MM -Á performed experiments and data analysis. LR and AC designed experiments and contributed to the manuscript writing. RT outlined the manuscript, overviewed the experiments, analyzed data, and wrote the manuscript.

## Conflict of Interest

The authors declare that the research was conducted in the absence of any commercial or financial relationships that could be construed as a potential conflict of interest.
